# Clinical Features of Cryptococcal Meningoencephalitis in HIV-Positive and -Negative Patients in a Resource-Limited Setting

**DOI:** 10.3390/jof9090869

**Published:** 2023-08-23

**Authors:** Rattagan Kajeekul, Pawut Mekawichai, Methee Chayakulkeeree

**Affiliations:** 1 Department of Internal Medicine, Maharat Nakhon Ratchasima Hospital, Nakhon Ratchasima 30000, Thailand; mekawichai@hotmail.com; 2 Division of Infectious Diseases and Tropical Medicine, Department of Medicine, Faculty of Medicine Siriraj Hospital, Mahidol University, Bangkok 10700, Thailand

**Keywords:** *Cryptococcus*, cryptococcosis, cryptococcal meningitis, cryptococcal meningoencephalitis, fungal infection, HIV

## Abstract

Cryptococcal meningoencephalitis is a systemic fungal infection in immunocompromised and immunocompetent individuals. This study investigated the clinical characteristics and factors associated with mortality in HIV-associated and non-HIV-associated cryptococcal meningoencephalitis in a resource-limited setting. This was a retrospective cohort study of patients with cryptococcal meningoencephalitis between January 2009 and December 2019 at a tertiary teaching hospital in Thailand. Overall, 1019 patients with cryptococcal meningoencephalitis were enrolled, and 923 (90.6%) were HIV-positive. The patients with HIV-associated cryptococcal meningoencephalitis were younger than the HIV-negative patients (37 versus 56 years, *p* < 0.01). The HIV-negative patients were more likely to have underlying conditions (52.1% versus 7.5%; *p* < 0.01), had a longer median duration of headaches prior to admission (14 days versus 6 days, *p* < 0.01), and were more likely to have an altered mental status at presentation (36.5% versus 18.6%, *p* < 0.01) and pulmonary involvement (15.6% versus 0.8%, *p* < 0.01). The HIV-positive patients had lower cerebrospinal fluid (CSF) white blood cell counts (4 versus 94 cells/mm^3^; *p* < 0.01), lower CSF protein (69 versus 157 mg/dL; *p* < 0.01), higher CSF glucose (38.8 versus 21 mg/dL; *p* < 0.01), and more frequent cryptococcemia (44.1% versus 20.5%; *p* < 0.01). The mortality rate was high but not significantly different between the two groups (30.2% versus 33.2%; *p* = 0.53). The HIV-positive patients with comorbidities, fever, an altered mental status at presentation, a CSF white blood cell count below 20 cell/mm^3^, fungemia, and positive CSF India ink were independently associated with 30-day mortality. In comparison, an altered mental status at presentation and fungemia were associated with 30-day mortality in HIV-negative patients. In conclusion, HIV-negative patients with cryptococcal meningoencephalitis had more extensive central nervous system inflammation, although the two groups’ mortality rates were similar. Unfavorable prognostic factors included comorbidities, fever, an altered mental status at presentation, a low CSF white blood cell count, fungemia, and positive CSF India ink.

## 1. Introduction

Cryptococcosis is a serious opportunistic infection caused by an encapsulated yeast of the *Cryptococcus* species with significant mortality and morbidity in individuals with advanced human immunodeficiency virus (HIV) infection [1]. The most common pathogenic *Cryptococcus* species are *Cryptococcus neoformans* and *Cryptococcus gattii* [2]. The prevalence of cryptococcosis in immunocompetent persons has increased. *C. neoformans* mainly causes invasive infection in immunocompromised patients, whereas *C. gattii* usually causes diseases in apparently immunocompetent individuals [2]. The most common manifestation of cryptococcosis is meningoencephalitis, followed by pulmonary cryptococcosis [2,3]. Cryptococcal meningoencephalitis can be found in healthy patients as well as in patients with immune dysfunction, such as organ transplantation, systemic lupus erythematosus, rheumatic disorders, hematologic malignancies, and those in receipt of immunosuppressive therapy [4,5,6,7,8,9,10,11]. Pulmonary cryptococcosis is recognized more often in individuals without HIV disease, whereas cryptococcal meningoencephalitis is the most common clinical presentation in patients with acquired immunodeficiency syndrome (AIDS) [12,13,14]. Recently, the prevalence of non-HIV-associated cryptococcal meningoencephalitis has increased in developed countries due to the increased use of immunosuppressive agents and the advanced treatment of cancer [15]. In contrast, the prevalence of HIV-associated cryptococcosis has reduced because of the widespread use of antiretroviral agents. However, few studies have reported comparative data on the clinical manifestations and mortality of HIV-associated and non-HIV-associated cryptococcal meningoencephalitis [16,17,18,19]. Previous studies have reported inconsistent results regarding the factors associated with patient outcomes, which have depended on the country, its socioeconomics, and hospital facilities for patient care. Furthermore, novel risk factors associated with systemic cryptococcosis, such as anti-granulocyte-macrophage colony-stimulating factor autoantibodies and small-molecule kinase inhibitors, especially Bruton tyrosine kinase inhibitors or Janus kinase inhibitors, have been identified [20,21]. Nevertheless, some patients with cryptococcal meningoencephalitis were apparently immunocompetent.

The treatment of cryptococcal meningoencephalitis in HIV patients comprises induction antifungal therapy with an amphotericin B product in combination with flucytosine for 1–2 weeks, followed by fluconazole [2]. There is no randomized controlled trial of cryptococcal meningoencephalitis in non-HIV patients. However, the combination of amphotericin B and flucytosine is recommended for 4–6 weeks of induction therapy [2]. In resource-limited countries such as Thailand, flucytosine was unavailable until July 2019. Therefore, before July 2019, we used amphotericin B monotherapy or amphotericin B in combination with fluconazole as an induction regimen. However, it has shown inferiority to amphotericin B plus flucytosine in several studies. In this study, we compared the clinical characteristics and outcomes in Thai patients who had cryptococcal meningoencephalitis with and without HIV infection, as well as the factors associated with 30-day mortality. The results of this study represent real-life practices and outcomes in resource-limited settings.

## 2. Materials and Methods

### 2.1. Study Design

This study was a 10-year retrospective cohort study using the electronic medical records of hospitalized patients aged more than 18 years who were diagnosed with cryptococcal meningoencephalitis from January 2009 to December 2019 at Maharat Nakorn Ratchasima Hospital, a tertiary referral teaching hospital in the northeast of Thailand. Cryptococcal meningoencephalitis was defined as one or more of the following: (1) having a positive cerebrospinal fluid (CSF) culture for *Cryptococcus* species, (2) having a positive CSF India ink preparation, and (3) having a positive CSF cryptococcal antigen. CSF cryptococcal antigen was identified using a commercial lateral flow assay (IMMY, Inc., Norman, OK, USA). *Cryptococcus* species were identified by morphology and biochemical tests.

### 2.2. Study Population and Data Collection

Eligible cases were identified from the ICD 10 codes by searching for codes B45.1 and B45.9, which diagnose cryptococcosis as a principal diagnosis or comorbidity. Patients with a clinical diagnosis of meningoencephalitis were screened, and the data of cerebrospinal fluid analyses were obtained. The cerebrospinal fluid results met the criteria compatible with defined cryptococcal meningoencephalitis, and patients aged ≥18 years were included in the study. The study data were collected and managed using electronic data capture tools. The data included age, sex, underlying diseases, initial clinical symptoms and signs, duration of symptoms (especially headache), fungemia, laboratory data, and clinical outcomes. CSF profiles including intracranial pressure, CSF cell counts, CSF proteins, CSF sugar, and CSF microbiological tests were also collected and analyzed. All subjects were divided into HIV-positive and HIV-negative groups. Data, including the baseline characteristics, clinical profiles, laboratory findings, outcomes, and 30-day mortality, were recorded. Subjects with inadequate medical data were excluded from the study.

The study was approved by the Research Ethics Committee of Maharat Nakhon Ratchasima Hospital, Nakhon Ratchasima, Thailand (IRB no. 063/2020).

### 2.3. Statistical Analysis

Categorical data were presented as numbers and percentages. Continuous data were expressed as means and standard deviations (SDs) or medians and ranges, based on the data distribution. Categorical variables were compared using the chi-square test or Fisher’s exact test. Numerical variables were compared using Student’s *t*-test or the Mann–Whitney rank-sum test. For multivariate analysis, parameters with a *p*-value less than 0.2 in univariate analysis were subjected to analysis by using a logistic regression model to identify independent factors associated with 30-day mortality. Statistical analyses were performed using SPSS Statistics, version 26.

## 3. Results

### 3.1. Demographics and Clinical Characteristics

From 2009 to 2019, 1019 patients were diagnosed with cryptococcal meningoencephalitis, of whom 923 (90.6%) were HIV-positive and 96 (9.4%) were HIV-negative. The median CD4 cell counts for the HIV-positive patients was 22 cells/mm^3^. The demographic data, clinical features, and mortality rates of HIV-positive and HIV-negative cryptococcosis are shown in Table 1.

The patients with HIV-associated cryptococcal meningoencephalitis were significantly younger (mean age: 37.4 years) than the HIV-negative patients (56.0 years). The patients with HIV-associated cryptococcal meningoencephalitis were significantly younger than the HIV-negative patients. The proportion of males to females was comparable between those with and without an HIV infection (68.7% and 65.6%, respectively). Comorbidities were documented in 50 patients (52.1%) in the HIV-negative group and 69 (7.5%) in the HIV-positive group, in which the HIV-negative patients had more comorbidities than the HIV-positive patients. Common comorbid diseases in the HIV-negative patients were hypertension, diabetes mellitus, and systemic lupus erythematosus. The mean headache duration before admission was 14 days for the HIV-negative patients and 6 days for the HIV-positive patients, respectively. The HIV-negative patients had a longer median duration of headaches prior to admission and were more likely to have an altered mental status at presentation than the HIV-positive patients (36.5% versus 18.6%, *p* < 0.01). In addition, the HIV-negative patients were more likely to have pulmonary involvement (15.6% versus 0.8%, *p* < 0.01), although the HIV-positive patients were more likely to have fungemia (44.1% versus 20.5%, *p* < 0.01). Other clinical signs and symptoms did not differ between those in both groups. These included fever, neurological manifestations, and respiratory symptoms. The 30-day mortality rates were also comparable between the HIV-positive and HIV-negative patients.

Patients with cryptococcal meningoencephalitis had increased intracranial pressure, but the mean initial levels of intracranial pressure were not different between the HIV-positive and HIV-negative individuals (27.0 cmH_2_O and 25.0 cmH_2_O, respectively). The HIV-negative cryptococcal meningoencephalitis patients had higher median CSF white blood cell counts (94.0 cells/mm^3^ versus 4.0 cells/mm^3^) and protein (157.0 mg/dL versus 69.0 mg/dL) than the HIV-positive patients, whereas the median CSF glucose (21.0 mg/dL versus 38.8 mg/dL) and mean CSF/blood glucose ratio (0.24 versus 0.33) were lower in the HIV-negative patients (Table 2). The rates of positive India ink preparation of the CSF (50.5% in the HIV-negative group and 66.1% in the HIV-positive group) and positive CSF fungal cultures (60.4% in the HIV-negative group and 71.0% in the HIV-positive group) tended to be higher in those with an HIV infection, but these differences were not statistically significant between the groups.

### 3.2. Outcomes and Mortality

The 30-day mortality was 30.2% (279 out of 923) for the HIV-positive patients and 33.2% (32 out of 96) for the HIV-negative patients (*p* = 0.53) (Table 1). In both HIV-associated and non-HIV-associated cryptococcal meningoencephalitis, the factors associated with 30-day and 90-day mortality included an age >50 years, comorbidities, an altered mental status at presentation, a CSF WBC < 20 cells/mm^3^, the presence of fungemia, and positive CSF India ink (Table 3 and Table 4).

We separately analyzed the factors associated with 30-day mortality for patients with HIV-associated and non-HIV-associated cryptococcal meningoencephalitis. An age of more than 50 years, comorbidities, fever, seizure, an altered mental status at presentation, high open pressure >18 cm H_2_O, a CSF white blood cell count <20 cells/mm^3^, CSF protein >45 mg/dL, a positive India ink preparation of the CSF, and fungemia were associated with mortality for HIV-positive cryptococcal meningoencephalitis patients. In HIV-negative patients, only an altered mental status at presentation and fungemia were associated with mortality (Table 5).

A multivariable binary logistic regression model included variables with statistically significant associations in univariate analysis with a *p*-value < 0.2. In the multivariate analysis, the 30-day mortality of HIV-positive patients was independently associated with comorbidities, fever, an altered mental status at presentation, a CSF white blood cell count below 20 cells/mm^3^, fungemia, and a positive CSF India ink preparation (Table 6). In HIV-negative patients, an altered mental status at presentation and fungemia were independently associated with mortality.

## 4. Discussion

To our knowledge, this is the largest study to compare the clinical characteristics of HIV-positive and HIV-negative cryptococcal meningoencephalitis. Although early HIV diagnosis and universal access to antiretroviral therapy have reduced the incidence of HIV-associated cryptococcal meningoencephalitis, it remains a common opportunistic infection [22,23]. In Thailand, cryptococcal meningoencephalitis is the third most common opportunistic infection, following tuberculosis and pulmonary pneumocystosis. However, cryptococcal diseases are increasingly reported in those without an HIV infection, partly because of the widespread use of immunosuppressive therapy for transplantation, cancer, and autoimmune disease [15]. Increased awareness to detect and diagnose cryptococcal diseases in non-HIV individuals is also associated with more case reports. Adult-onset immunodeficiencies, such as anti-interferon-gamma autoantibodies syndrome and anti-granulocyte-macrophage colony-stimulating factor (anti-GM-CSF) autoantibodies syndrome, have been recognized as a risk factor of non-HIV-associated cryptococcosis. The treatment of cryptococcal meningoencephalitis comprises induction, consolidation, and maintenance antifungal therapy. Induction antifungal treatment is the most crucial step to eradicate cryptococci from the CSF. An amphotericin B product in combination with flucytosine is recommended for induction antifungal therapy to treat cryptococcal meningoencephalitis [2,14]. In a resource-limited setting such as Thailand, where flucytosine was not available during this study, the treatment outcomes of patients with cryptococcal meningoencephalitis might differ from those of patients in other countries. Flucytosine exhibits excellent penetration into the blood–brain barrier, and it has been shown to have a synergistic fungicidal activity with *C. neoformans* when combined with amphotericin B. Therefore, induction treatment without flucytosine may result in unfavorable outcomes. In this study, we found that the mortality of HIV-associated and non-HIV-associated cryptococcal meningoencephalitis is approximately 30%, which is extremely high compared with 11–20% in developed countries. A lack of flucytosine during induction therapy and inadequate supportive treatment, especially therapeutic lumbar puncture, may be attributable to the high mortality rates. Consistent with other studies, this study found that 58 (60.4%) patients with HIV-negative cryptococcal meningoencephalitis had underlying medical conditions, including hypertension, diabetes mellitus, or systemic lupus erythematosus [5,24]. During this study, we did not have a facility in which to perform autoantibody assays for anti-interferon-gamma and anti-GM-CSF. In our recent unpublished cohort study, these autoimmune diseases were found in approximately 30–40% of non-HIV-associated cryptococcosis cases in Thailand. Other studies have reported that the most common underlying medical condition in HIV-negative patients was receiving immunosuppressive agents for >2 weeks or prednisolone therapy of more than 700 mg [4,25,26].

Similar to other reports, we observed that in HIV-positive and HIV-negative individuals, men were more often infected by *Cryptococcus* [4,27,28,29], presumably because of the effects of testosterone. According to McClelland et al. [30], male patients were more likely to have severe cryptococcal meningoencephalitis because of interactions between *C. neoformans* and testosterone, which resulted in increased capsular glucuronoxylomannan release. Furthermore, macrophages in women phagocytosed *C. neoformans* more effectively than in men, and macrophages in male patients were more likely to be killed by *C. neoformans* rather than to kill *C. neoformans*. However, further research is warranted to explain these findings fully.

In our study, consistent with another report, the most common extracranial site of infection in HIV-negative patients was the respiratory system [24]. Pulmonary cryptococcosis in HIV-negative patients often presents with one or more pulmonary nodules or masses in chest radiographs. Almost all non-HIV patients with pulmonary cryptococcosis are asymptomatic, and sometimes it was provisionally diagnosed as lung cancer or tuberculosis. A diagnosis of pulmonary cryptococcosis usually requires a tissue biopsy or lobectomy [31]. Individuals with cryptococcal meningoencephalitis commonly present with a subacute or chronic course. Non-HIV-associated meningoencephalitis sometimes mimics metastatic cancer and may present with impaired cognitive function or dementia. In our study, HIV-positive patients had a shorter duration from symptom onset to diagnosis (median: 6 versus 14 days), similar to an earlier report [32]. Compared with HIV-positive patients, cryptococcal meningoencephalitis in HIV-negative patients tended to have a higher mortality rate, which may be caused by delayed diagnosis related to the subacute nature of symptom development [4,33].

We observed that HIV-negative patients had significantly higher numbers of CSF leukocytes, higher CSF protein, and lower CSF glucose compared with HIV-positive patients. This observation may reflect an aggressive inflammatory response in HIV-negative cryptococcosis [34]. The reported mortality rate of cryptococcal meningoencephalitis varies between 9% and 31% [5,16,35]. In our study, the mortality rates were high in both groups but not statistically significant (30.2% versus 33.2%).

The adverse prognostic factors for cryptococcal meningoencephalitis reported in previous studies included age < 35 years, an altered mental status, comorbidities, T-cell-suppressed patients, CSF glucose < 40 mg/dL, a CSF cell count < 20 cell/mm^3^, CSF antigen titer > 1:1024, positive CSF India ink, and brain abnormalities in computed tomography [29,36,37,38,39,40,41]. Through the multivariate analysis of our study, we found that the negative prognostic factors that were associated with 30-day and 90-day mortalities included age > 50 years, comorbidities, an altered mental status at presentation, a CSF WBC < 20 cells/mm^3^, fungemia, and positive CSF India ink. Compared with previous studies, there is consistent evidence showing that a high fungal burden with a low CSF immune response is associated with poor outcomes. However, we found that older patients had poorer prognoses, which is different from previous studies. This observation may be partly explained by the higher proportion of older non-HIV patients and may be linked to higher mortality. However, this finding needs to be explored further.

We performed a subgroup analysis of HIV-associated and non-HIV-associated cryptococcal meningoencephalitis. We found that age was not associated with mortality. However, an altered mental status and fungemia were associated with mortality in HIV-negative individuals, suggesting the presence of clinically significant immunosuppression in those patients. In contrast, in HIV-positive patients, comorbidities, severe disease (including fungemia), a high CSF fungal load, and low CSF white blood cells were associated with death. This may be explained partly by the unavailability of flucytosine used in combination with amphotericin B during the induction of antifungal therapy. All patients received amphotericin B with or without fluconazole as an induction treatment. Flucytosine was not available in Thailand until July 2019. However, inadequate supportive care, including intracranial pressure management and the delayed diagnosis and treatment of advanced HIV disease, may be other factors associated with mortality.

A strength of our study is that it had the largest sample size reported in a study of cryptococcal meningoencephalitis. However, this study had some limitations. First, this was a retrospective study using patient medical records and laboratory data. Second, we did not confirm the species of *Cryptococcus*, which may have impacted some outcomes because of differences in *C. neoformans* and *C. gattii* infections [42,43]. Third, the cryptococcal antigen was examined semi-quantitatively, whereas a quantitative report may be more accurate in indicating prognosis. Last, we could not evaluate the treatment’s exact duration because some patients were referred to community hospitals.

## 5. Conclusions

In summary, patients with HIV-positive and HIV-negative cryptococcal meningoencephalitis had different clinical manifestations and CSF findings. HIV-associated cryptococcal meningoencephalitis patients had fewer CSF leukocytes, a higher CSF/blood glucose ratio, and lower CSF protein, possibly related to a diminished immune response. The mortality of HIV-positive and HIV-negative cryptococcal meningoencephalitis was relatively high compared with other studies in Southeast Asia.

## Figures and Tables

**Table 1:** Clinical characteristics and mortality of HIV-positive and HIV-negative patients with cryptococcal meningoencephalitis (N = 1019).

Characteristics	HIV-Positive *n* = 923 (%)	HIV-Negative *n* = 96 (%)	*p*-Value
Mean age, years ± SD	37.4 ± 10.0	56.0 ± 15.2	<0.01
Female sex, *n* (%)	289 (31.3)	33 (34.4)	0.54
Comorbid disease, *n* (%) *	69 (7.5)	50 (52.1)	<0.01
Hypertension	23 (2.5)	18 (18.8)	<0.01
Diabetes mellitus	8 (0.9)	10 (10.4)	<0.01
Systemic lupus erythematosus	2 (0.2)	12 (12.5)	<0.01
Others	42 (4.6)	46 (16.7)	<0.01
Extra-central nervous system involvement, *n* (%)			
Pulmonary involvement	7 (0.8)	15 (15.6)	<0.01
Lymphadenitis	6 (0.7)	2 (2.1)	0.43
Fungemia	369 (44.1)	18 (20.5)	<0.01
Median duration of headache before admission, days (range)	6.0 (0–180)	14.0 (0–120)	<0.01
Alteration of consciousness at presentation, *n* (%)	172 (18.6)	35 (36.5)	<0.01
Clinical manifestations, *n* (%)			
Fever	618 (67)	68 (70.8)	0.44
Seizure	125 (13.5)	15 (15.6)	0.57
Visual disturbance	22 (2.4)	2 (2.1)	0.85
Cranial nerve palsy	37 (4)	5 (5.2)	0.57
Cough	23 (2.5)	5 (5.2)	0.12
Dyspnea	17 (1.8)	4 (4.2)	0.13
Stiffness of neck	419 (45.4)	46 (47.9)	0.64
Pupil edema	15 (1.6)	2 (2.1)	0.74
Weakness	22 (2.4)	5 (5.2)	0.10
Mortality rate at 30 days, *n* (%)	279 (30.2)	32 (33.2)	0.53

* One person may have more than one underlying disease. Abbreviations: SD, standard deviation; HIV, human immunodeficiency virus.

**Table 2:** Cerebrospinal fluid profile of HIV-positive and HIV-negative patients with cryptococcal meningoencephalitis.

Characteristics	HIV-Positive *n* = 923	HIV-Negative *n* = 96	*p*-Value
Median open pressure, mmH_2_O (range)	27.0 (10–75)	25.0 (6–60)	0.54
Median CSF white blood cell counts, cells/mm^3^ (range)	4.0 (0–2050)	94.0 (0–650)	<0.01
Median CSF glucose, mg/dL (range)	38.8 (0–169)	21.0 (1–122)	<0.01
Mean CSF/blood glucose ratio ± SD	0.33 ± 0.17	0.24 ± 0.19	<0.01
Median CSF protein, mg/dL (range)	69.0 (1.4–2240)	157.0 (30–787)	<0.01
Positive CSF India ink, *n* (%)	609 (66.1)	48 (50.5)	0.03
Positive CSF cryptococcal antigen, n (%)	857 (97.7)	89 (97.8)	0.90
Positive CSF culture for *Cryptococcus* spp., n (%)	654 (71.0)	58 (60.4)	0.34

Abbreviations: SD, standard deviation; HIV, human immunodeficiency virus; CSF, cerebrospinal fluid.

**Table 3:** Univariate and multivariate analyses of factors associated with 30-day mortality of patients with cryptococcal meningoencephalitis.

Characteristics	Univariate Analysis			Multivariate Analysis		
	Odds Ratio	95% CI	*p*-Value	Odds Ratio	95% CI	*p*-Value
Age > 50 years	1.63	1.12–2.33	0.008	2.09	1.40–3.11	<0.001
Female sex	1.23	0.93–1.63	0.16			
HIV infection	0.87	0.55–1.36	0.53			
Comorbidities	1.90	1.33–2.73	<0.001	1.64	1.09–2.47	0.017
Duration of headache > 14 days	0.69	0.47–1.01	0.56			
Fever	1.37	1.02–1.84	0.034			
Seizure	1.69	1.17–2.44	0.005			
Altered mental status at presentation	3.27	2.39–4.45	<0.001	2.96	2.10–4.18	<0.001
Stiffness of neck	1.09	0.84–1.43	0.514			
Open pressure > 18 mm H_2_O	1.30	0.95–1.78	0.98			
CSF WBC < 20 cells/mm^3^	1.64	1.25–2.15	<0.001	1.70	1.28–2.26	<0.001
CSF glucose < 40 cells/mm^3^	1.16	0.88–1.52	0.30			
CSF protein > 45 cells/mm^3^	1.39	1.04–1.87	0.026			
Fungemia	1.86	1.42–2.44	<0.001	1.80	1.10–2.93	0.018
Positive CSF culture for *Cryptococcus* spp.	1.06	0.79–1.42	0.69			
Positive CSF India ink	1.66	1.24–2.22	0.001	1.42	1.03–1.94	0.032

**Table 4:** Univariate and multivariate analyses of factors associated with 90-day mortality of patients with cryptococcal meningoencephalitis.

Characteristics	Univariate Analysis			Multivariate Analysis		
	Odds Ratio	95% CI	*p*-Value	Odds Ratio	95% CI	*p*-Value
Age > 50 years	2.11	1.48–2.99	<0.001	2.09	1.40–3.11	<0.001
Female sex	1.21	0.93–1.59	0.153			
HIV infection	0.90	0.59–1.37	0.62			
Comorbidities	2.12	1.48–3.03	<0.001	1.64	1.09–2.47	0.017
Duration of headache > 14 days	0.67	0.48–0.96	0.026			
Fever	1.51	1.15–1.98	0.003			
Seizure	1.75	1.22–2.50	0.002			
Altered mental status at presentation	3	2.19–4.13	<0.001	2.96	2.10–4.18	<0.001
Stiffness of neck	1.13	0.88–1.45	0.35			
Open pressure > 18 mm H_2_O	1.25	0.94–1.66	0.130			
CSF WBC < 20 cells/mm^3^	1.55	1.21–1.99	0.001	1.70	1.28–2.26	<0.001
CSF glucose < 40 cells/mm^3^	1.18	0.92–1.52	0.198			
CSF protein > 45 cells/mm^3^	1.26	0.96–1.67	0.102			
Fungemia	1.80	1.39–2.31	<0.001	1.80	1.10–2.93	0.018
Positive CSF culture for *Cryptococcus* spp.	0.98	0.75–1.29	0.91			
Positive CSF India ink	1.48	1.14–1.92	0.004	1.42	1.03–1.94	0.032

**Table 5:** Univariate analysis of factors associated with 30-day mortality of patients with HIV-associated and non-HIV-associated cryptococcal meningoencephalitis.

Factor	HIV-Positive			HIV-Negative		
	Odds Ratio	95% CI	*p*-Value	Odds Ratio	95% CI	*p*-Value
Age > 50 years	1.74	1.10–2.76	0.02	1.80	0.70–4.64	0.22
Female sex	1.23	0.91–1.65	0.18	1.23	0.51–2.98	0.65
Comorbidities	2.18	1.39–3.41	<0.01	1.71	0.70–4.19	0.24
Duration of headache > 14 days	0.70	0.46–1.07	0.09	0.56	0.22–1.43	0.22
Fever	1.40	1.03–1.91	0.03	1.08	0.42–2.76	0.87
Seizure	1.72	1.17–2.54	<0.01	1.41	0.45–4.38	0.55
Altered mental status at presentation	3.26	2.32–4.59	<0.01	3.56	1.46–8.67	<0.01
Stiffness of neck	1.10	0.83–1.45	0.53	1.07	0.46–2.49	0.89
Pulmonary involvement	0.92	0.18–4.79	0.92	0.69	0.20–2.36	0.55
Open pressure > 18 cm H_2_O	1.45	1.04–2.02	0.03	0.49	0.18–1.29	0.14
CSF WBC < 20 cells/mm^3^	1.75	1.31–2.34	<0.01	1.31	0.48–3.57	0.60
CSF glucose < 40 cells/mm^3^	1.10	0.82–1.45	0.55	2.12	0.76–5.93	0.15
CSF protein > 45 cells/mm^3^	1.48	1.09–2.00	0.01	0.38	0.43–3.40	0.37
Fungemia	1.76	1.32–2.33	<0.01	5.57	1.94–16.0	<0.01
Positive CSF culture for *Cryptococcus* spp.	1.09	0.80–1.48	0.60	0.94	0.39–2.23	0.88
Positive CSF India ink	1.81	1.33–2.48	<0.01	0.97	0.41–2.27	0.94

**Table 6:** Multivariate analysis of factors associated with 30-day mortality of patients with HIV-associated and non-HIV-associated cryptococcal meningoencephalitis.

Factor	HIV-Positive		HIV-Negative	
	Adjusted Odds Ratio (95% CI)	*p*-Value	Adjusted Odds Ratio (95% CI)	*p*-Value
Comorbidities	1.85 (1.12–3.06)	0.02		
Fever	1.42 (1.02–1.98)	0.04		
Altered mental status at presentation	3.53 (2.42–5.17)	<0.01	3.30 (1.28–8.47)	0.01
CSF WBC < 20 cells/mm^3^	1.59 (1.13–2.24)	<0.01		
Fungemia	1.44 (1.04–1.97)	0.03	5.17 (1.73–15.50)	<0.01
Positive CSF India ink	1.55 (1.08–2.22)	0.02		

## Data Availability

All other study data are included in the article, further inquiries can be directed to the corresponding authors.
